# Enhancement in Electrical and Thermal Properties of LDPE with Al_2_O_3_ and h-BN as Nanofiller

**DOI:** 10.3390/ma15082844

**Published:** 2022-04-13

**Authors:** Lijuan He, Zhanzheng Ye, Junji Zeng, Xiong Yang, Dawei Li, Xiangyu Yang, Yu Chen, Yuewu Huang

**Affiliations:** 1 College of Science, Harbin University of Science and Technology, Harbin 150080, China; yzz2469592133@163.com (Z.Y.); zjj941122@163.com (J.Z.); deardawei4li@hrbust.edu.cn (D.L.); xiangyu_yang1995@163.com (X.Y.); cy374345509@163.com (Y.C.); wuyuehuang@gmail.com (Y.H.); 2 Key Laboratory of Engineering Dielectric and Its Application, Ministry of Education, Harbin University of Science and Technology, Harbin 150080, China; 3 State Key Laboratory of Electrical Insulation and Power Equipment, School of Electrical Engineering, Xi’an Jiaotong University, Xi’an 710049, China; xiongyang221@stu.xjtu.edu.cn

**Keywords:** hexagonal boron nitride, alumina fiber, low-density polyethylene, space charge

## Abstract

Low-density polyethylene (LDPE) has excellent dielectric properties and is extensively used in electrical equipment. Hexagonal boron nitride (h-BN) is similar to a graphite-layered structure, and alumina fiber (Al_2_O_3_) has high-temperature resistance and a strong performance. Herein, we prepared Al_2_O_3_-h-BN/LDPE nanocomposites by using LDPE as the matrix material and h-BN and Al_2_O_3_ as the fillers. The influence of different doping contents and the mass ratio of Al_2_O_3_ and h-BN (1:1) to LDPE on the electrical properties and thermal conductivity of the nanocomposites was examined. The results showed that the suppression effect on space charge was the most obvious and average. The charge density was the lowest and had the minimum decay rate when the doping content was 2%. The breakdown strength of the film reached the maximum value of 340.1 kV/mm, which was 12.3% higher than that of the pure LDPE (302.8 kV/mm). The thermal diffusivity of the composite sample was also higher than that of the single h-BN-doped sample when the content of h-BN and Al_2_O_3_ was 7%. The thermal conductivity was 59.3% higher than that of the pure LDPE sample and 20% higher than that of h-BN/LDPE.

## 1. Introduction

HVDC cables are widely used in the energy, communication, and petrochemical industries. However, the service life of HVDC cables is often greatly reduced owing to electrical aging and thermal aging during the transmission process [1]. Therefore, exploring a way to make the cable exhibit excellent electrical and thermal properties is a key issue.

Low-density polyethylene (LDPE) is extensively applied in HVDC cables owing to its good physical and chemical properties [2], but its low thermal conductivity (about 0.32 W/(m·K)) limits this application [3]. Thermal conductivity is a parameter that represents the heat transfer per unit area per unit time. Nanofiller doping can greatly improve the polymer’s electrical, thermal, mechanical, and rheological properties. The introduction of nanofiller boron nitride (BN)/alumina fiber (Al_2_O_3_) can greatly improve the thermal conductivity and electrical properties of the polymer. Zhang et al. [4] found that BN nanosheets/styrene–(ethylene-co-butylene)–styrene triblock copolymer/polypropylene (BNNS/SEBS/PP) composite insulation prepared with multilayer hot-pressing materials can significantly improve the electrical insulation and thermal conductivity of composites. Du et al. [5] studied the effect of thermal conductivity for different mass fractions of silicone rubber (SiR)/hexagonal BN (h-BN) on its erosion resistance. They found that the thermal conductivity and arc resistance of the composite significantly improve with the increased mass fraction of h-BN fillers [6]. The thermal conductivity of α-Al_2_O_3_ epoxy resin composites wrapped by BN-doped nanosheets is also much higher than that of Al_2_O_3_-doped composites. Fei Chuan et al. [7] prepared alumina-modified polyimide thermally conductive composite films by in situ polymerization. They found that the thermal conductivity of polyimide composite films after adding 20% and 30% micron alumina is 1.5 and 2.4 times that of pure polyimide films, respectively, and that polyimide composites modified with alumina have an excellent thermal stability.

Although many experts and scholars have conducted substantial research on BN/Al_2_O_3_-doped composites and their electrical properties and thermal conductivity, few studies have been performed on mixing nanofillers with excellent thermal conductivity. In the present study, LDPE doped with h-BN and Al_2_O_3_ fibers were used as fillers to study the effects of two fillers with different structures, types, and dimensions on the thermal conductivity and electrical properties of LDPE.

## 2. Experimental

### 2.1. Materials and Reagents

LDPE (type 18D) has a density of 0.918 g/cm^3^ and a melting point of 110–125 °C. The LDPE used in this study was produced by Daqing Petrochemical in Heilongjiang, China. The particle diameters of the h-BN with a density of 2.3 g/cm^3^ and Al_2_O_3_ with a density of 3.9 g/cm^3^ were both 50 nm. Both kinds of nanoparticles were produced by Qinghe County Kegong Metallurgical Materials Co., Ltd., Handan, China.

### 2.2. Sample Preparation

First, nano-BN and Al_2_O_3_ were mixed by a KH550 coupling agent at a mass ratio of 1:1 and then filtered and dried to form a filler. Different contents of fillers and LDPE were melt blended in a Happ RM-200A mixing torque rheometer to prepare masterbatches with different mass fractions. The masterbatches with different mass fractions were cut into small pellets and dried for 24 h before being pressed into samples of different thicknesses with a hot-pressing flat vulcanizing machine for tests. Pressurization was completed when the pressure was 25 MPa. After cooling with circulating water, the composite film to be tested was obtained. The temperature of the entire pressurization process was set to 120 °C. The composite films with different mass fractions were tested by coating a layer of aluminum film on both sides of the film with a vacuum-coating machine. To simplify the description, the content of pure LDPE, nano-h-BN particles, and Al_2_O_3_ fibers were denoted as A, B, and C, respectively. The samples with different contents are shown in Table 1.

### 2.3. Analytical Methods

A Quanta200 scanning electron microscopy (SEM) system from Philips (Royal Philips Electronics, Amsterdam, The Netherlands) was used to observe the dispersion of nano-h-BN particles and Al_2_O_3_ fibers in the LDPE matrix and the microscopic morphology of the nanocomposite films.

An Empyrean X-ray diffraction (XRD) instrument from PANalytical Corporation (Almelo, The Netherlands), with phase analysis and comparison, was used to examine the crystalline structure of the LDPE after adding nanofillers.

A new EQUINOX-50 Fourier transform infrared (FTIR) spectrometer from Bruker (Bremen, Germany) was used to test the infrared spectra of the pure LDPE and its composite films.

A Concept40 broadband dielectric spectrometer from Novocontrol (Montabaur, Germany) was used to measure the dielectric properties of the pure LDPE and Al_2_O_3_-h-BN/LDPE samples at room temperature.

The BDJC power frequency breakdown-test platform of Beiguang Precision Instrument Co., Ltd. (Beijing, China) was used to test the breakdown strength of the pure LDPE and composite films with different nanodoping contents at room temperature. The diameter of the sample was 60 mm and the thickness was 50 μm.

An electroacoustic pulse device independently developed by Shanghai Jiaotong University, Shanghai, China, was used to test the space-charge distribution of the sample at room temperature. Before the test, a small amount of silicone oil was applied onto the contact surface of the semiconductor, the sample, and the aluminum electrode to prevent the formation of an air gap, which can interrupt signal transmission, between the sample and electrode.

An LFA447 laser thermal-conductivity meter manufactured by NETZSCH (Selb, Germany) was used to measure the thermal diffusivity and calculate the thermal conductivity of the single-doped nano-h-BN filler and the two-doped nanofiller h-BN and Al_2_O_3_. The test temperature range of this instrument was 25–300 °C, the thermal diffusivity range was 0.1–1000 mm^2^/s, and the sample was 1.26 cm in diameter and 1–1.5 mm thick. The thermal conductivity was determined according to the following Equation:

(1) λ(T)=α(T)×Cp(T)×ρ(T)

where *T* is a certain temperature; λ(T) is the thermal conductivity; α(T) is the thermal diffusivity; Cp(T) is the specific heat capacity of the sample; and ρ(T) is the sample density.

## 3. Results and Discussion

### 3.1. Microstructure Characterization

**Figure 1:**
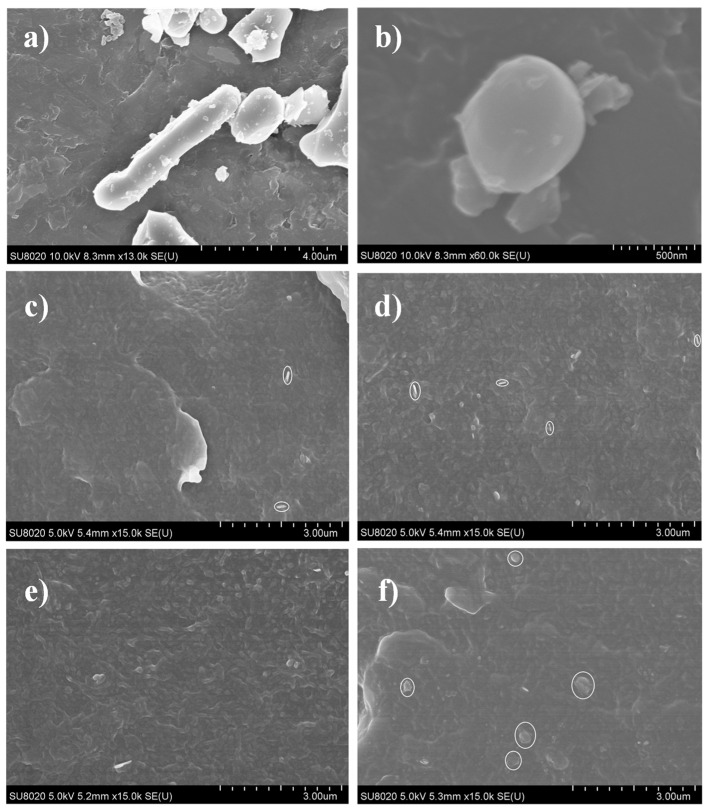
Shows the SEM images of Al_2_O_3_, BN, and Al_2_O_3_-h-BN/LDPE composite film: (**a**) Al_2_O_3_; (**b**) BN; (**c**) 1 wt%; (**d**) 2 wt%; (**e**) 4 wt%; and (**f**) 7 wt%.

Figure 1 shows the SEM images of the Al_2_O_3_, BN, and Al_2_O_3_-h-BN/LDPE composite film. According to Figure 1a,b, the length of alumina fiber is about 5.21 microns, and the diameter of spherical BN is about 780 nanometers. It can be seen in Figure 1c–e that the nanofiller Al_2_O_3_-h-BN is uniformly dispersed in the composite film in the presence of the coupling agent KH500 [8]. When the filler was 1 wt%, 2 wt%, and 4 wt%, it showed a good dispersibility. As shown in Figure 1f, for 7 wt%, there was an obvious agglomeration at the marked circle in the cross-sectional view of the Al_2_O_3_-h-BN/LDPE composite film.

**Figure 2:**
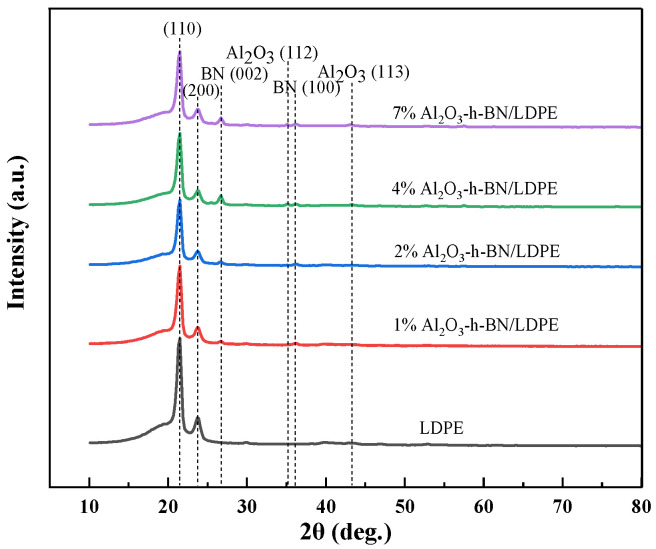
XRD spectra of pure LDPE and its Al_2_O_3_-h-BN/LDPE nanocomposite specimens.

Figure 2 shows the XRD patterns of the composite samples with different contents. With the increased content of the nanofillers h-BN and Al_2_O_3_, prominent diffraction peaks appeared at 26.6°, 36.6°, 35.2°, and 43.3°. These peaks represent that crystalline form diffraction occurred on the crystal plane, corresponding with the crystal planes of 002, 100, 112, and 113 of the crystal form. As the LDPE content gradually decreased, the diffraction peaks corresponding with the diffraction angles of crystal planes 110 and 200 at 21.3° and 23.6° also gradually decreased. By comparing the characteristic peaks before and after doping with the two nanofillers, it can be seen that no difference existed between the diffraction crystal planes and the standard crystal planes of LDPE, h-BN, and Al_2_O_3_ in the composite film. Meanwhile, the composite film did not produce impurity during the melt-blending and hot-pressing stages. Thus, adding the two nanoparticles did not change the crystalline structure of the LDPE.

**Figure 3:**
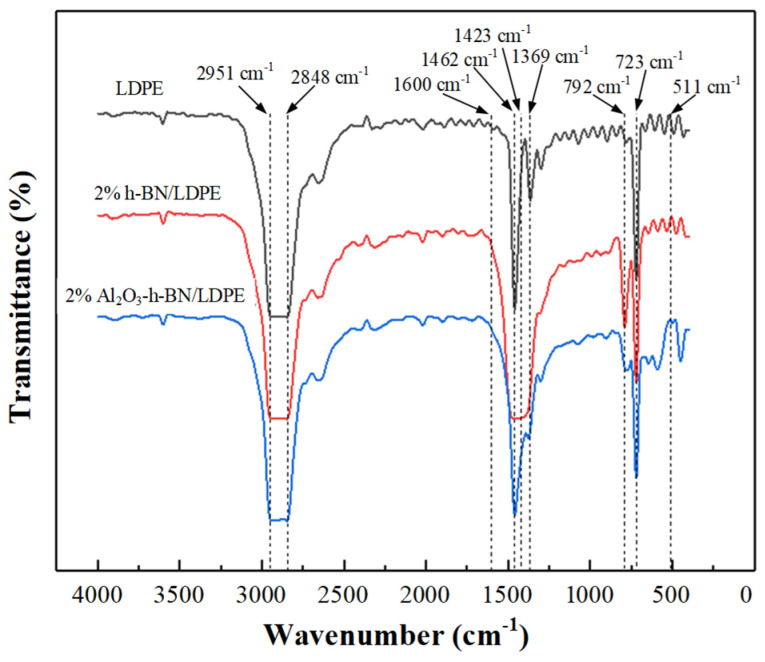
The infrared-light spectrum of pure LDPE and its nanocomposite films.

To study the chemical-bonding state of the composite materials formed by h-BN, Al_2_O_3,_ and LDPE, FTIR spectra were obtained, as shown in Figure 3. Composite films and pure LDPE were analyzed. LDPE had strong absorption peaks at 1462, 1369, and 723 cm^−1^, and these three absorption peaks corresponded with the in-plane bending shear vibration peaks, out-of-plane bending swing vibration peak, and swing vibration peak of C-H in LDPE [9]. The characteristic peak at 3608 cm^−1^ was the O-H stretching vibration peak formed by the absorption of moisture in the air [10]. Compared with the absorption peaks of pure LDPE, the corresponding peaks of h-BN/LDPE at 1423 and 792 cm^−1^ were B-N stretching vibration and deformation vibration absorption peaks, respectively. Comparing the composites formed by the two nanofillers, h-BN and Al_2_O_3,_ and LDPE with pure LDPE and h-BN/LDPE, the peak at 1462 cm^−1^ was the bonding after adding Al_2_O_3_. The broad absorption band appearing at 511 cm^−1^ was the vibration absorption peak of Al-O, belonging to the characteristic absorption peak of alumina [11]. These results show that adding nano-h-BN and Al_2_O_3_ fibers did not destroy the structure of LDPE molecular chains.

### 3.2. Dielectric Property Testing

**Figure 4:**
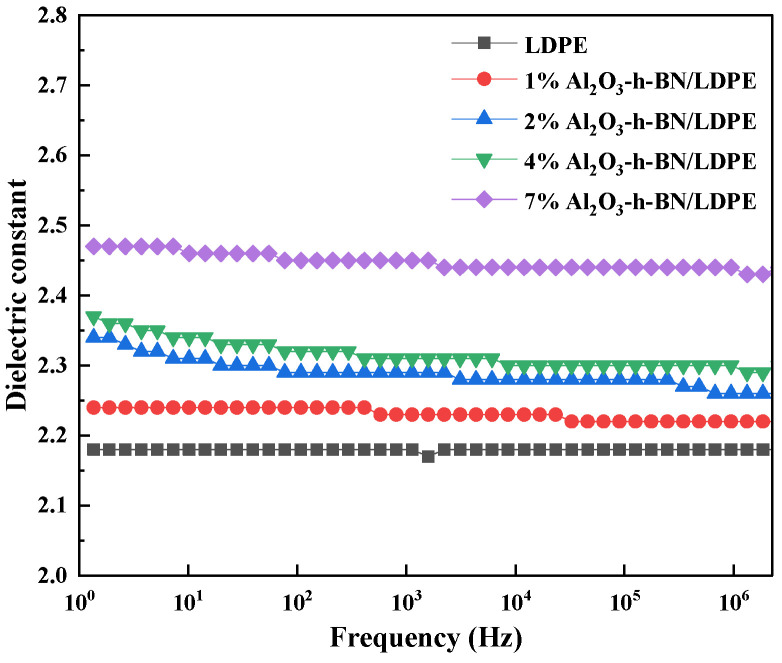
Plot of dielectric permittivity of LDPE and its nanocomposite specimens at room temperature.

The relationship between the relative permittivity and frequency of Al_2_O_3_-BN/LDPE composite films with different doping contents and pure LDPE at room temperature (25 °C) is shown in Figure 4. With increased frequency, the relative dielectric permittivity of the pure LDPE remained basically unchanged at 2.18. With the addition of h-BN and Al_2_O_3_, the relative permittivity [12] gradually increased, and the relative permittivity of the dopant content of 2 wt% and 4 wt% did not significantly differ. When the doping content was 7 wt%, the relative permittivity increased by 0.29 at the maximum and 0.25 at the minimum at 10–10^6^ Hz, which had little effect on the low permittivity of the LDPE [13]. This finding indicates that adding nano-h-BN and Al_2_O_3_ did not change the low dielectric permittivity properties of the LDPE. There are a large number of organic and inorganic interfaces in nanocomposites, and interface polarization exists when the frequency is low. However, the interface polarization is difficult to form when the frequency is high, so the dielectric permittivity decreases.

**Figure 5:**
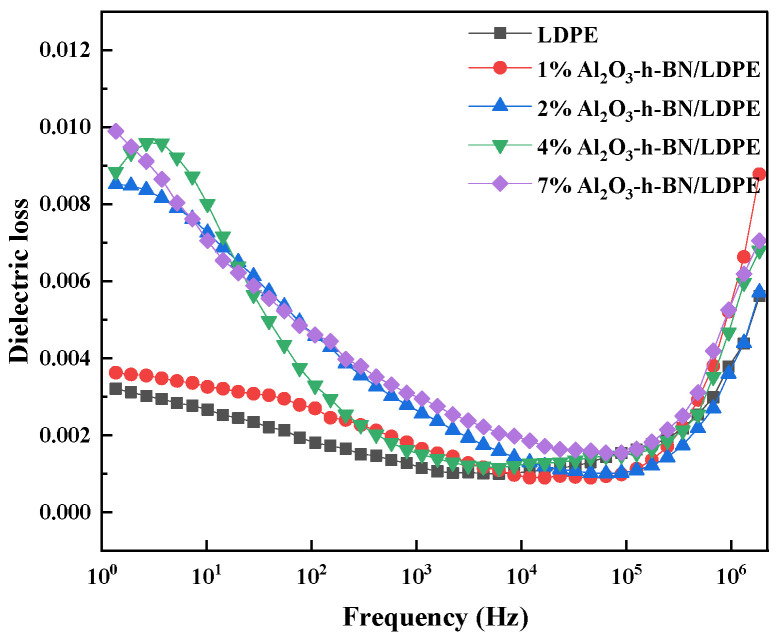
Dielectric loss of LDPE and Al_2_O_3_-h-BN/LDPE composite specimens as a function of frequency.

Figure 5 shows the relationship between the dielectric loss and frequency of LDPE and Al_2_O_3_-h-BN/LDPE composite samples. Adding nanofillers increased the dielectric loss [14], but the dielectric loss was only 0.0074 higher than that of the pure LDPE when the doping content was 7 wt% by weight. The dielectric loss of the pure LDPE and nanocomposite films doped with different contents initially decreased and then increased with increased frequency from 10 to 10^6^, and the difference in the dielectric loss at 10^5^ Hz was the smallest, i.e., only about 0.001. Apparently, the dielectric loss of the composite was only slightly affected by the h-BN and Al_2_O_3_ fibers. So, the application of its insulating properties in cables was hardly affected. Dielectric loss mainly includes conductance loss and polarization loss. At low frequency, the electric field changes slowly, and the polarization of dielectric material is basically synchronous with the change of the external electric field, so the polarization loss is low. However, at high frequencies, the polarization of the polymer film cannot synchronize with the change of the applied electric field, and the dipole composed of positive and negative charges will tend to align along the direction of the electric field, and gradually begin to rotate to produce a polarization loss [15].

**Figure 6:**
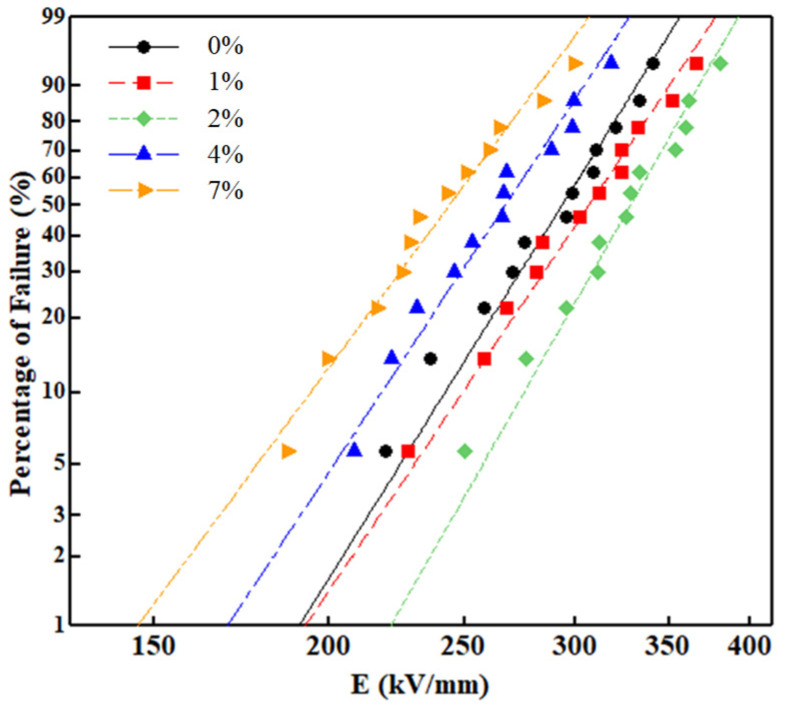
Weibull distribution of breakdown field intensity of pure LDPE and its nanocomposite specimens.

Figure 6 and Table 2 show the Weibull distribution of the breakdown field strength and the Weibull distribution parameters of the LDPE and nanocomposite samples [16]. Twelve tests were performed on the LDPE and its nanocomposite samples, respectively, and the DC breakdown strength of the pure LDPE was 302.4 kV/mm. The breakdown field strength of the Al_2_O_3_-h-BN/LDPE nanocomposite films initially increased and then decreased with an increased mass fraction of Al_2_O_3_ fibers and h-BN particles. When the mass fraction of nanofillers was 2 wt%, the breakdown field strength reached the maximum value of 340.1 kV/mm. When the mass fraction of nanofillers was 4 wt% and 7 wt%, the DC breakdown field strength was lower than that of the pure LDPE. The DC breakdown field strength of the composite films was 5.54% and 12.3% higher than that of the pure LDPE when the doping amount was 1 wt% and 2 wt%, respectively. In summary, Al_2_O_3_ and h-BN had a good dispersion in the LDPE when doped with a lower content of nanomaterials [17], and more deep traps were introduced into the LDPE, thereby reducing the carrier concentration and mobility in the matrix material [18], Therefore, the local electric field is alleviated, and the breakdown strength of the composite is eventually improved due to the significant reduction of space charge. When the nanodoping content was too high at 4 wt% and 7 wt%, the dispersion of nanoparticles in the LDPE was poor and the aggregation of nanoparticles was relatively large. The agglomeration of particles may become defects, which aggravate the electric field distortion around particles and lead to the partial discharge of the sample, thus greatly reducing the breakdown field strength of the Al_2_O_3_-h-BN/LDPE composite film.

### 3.3. Space-Charge Distribution Measurement

**Figure 7:**
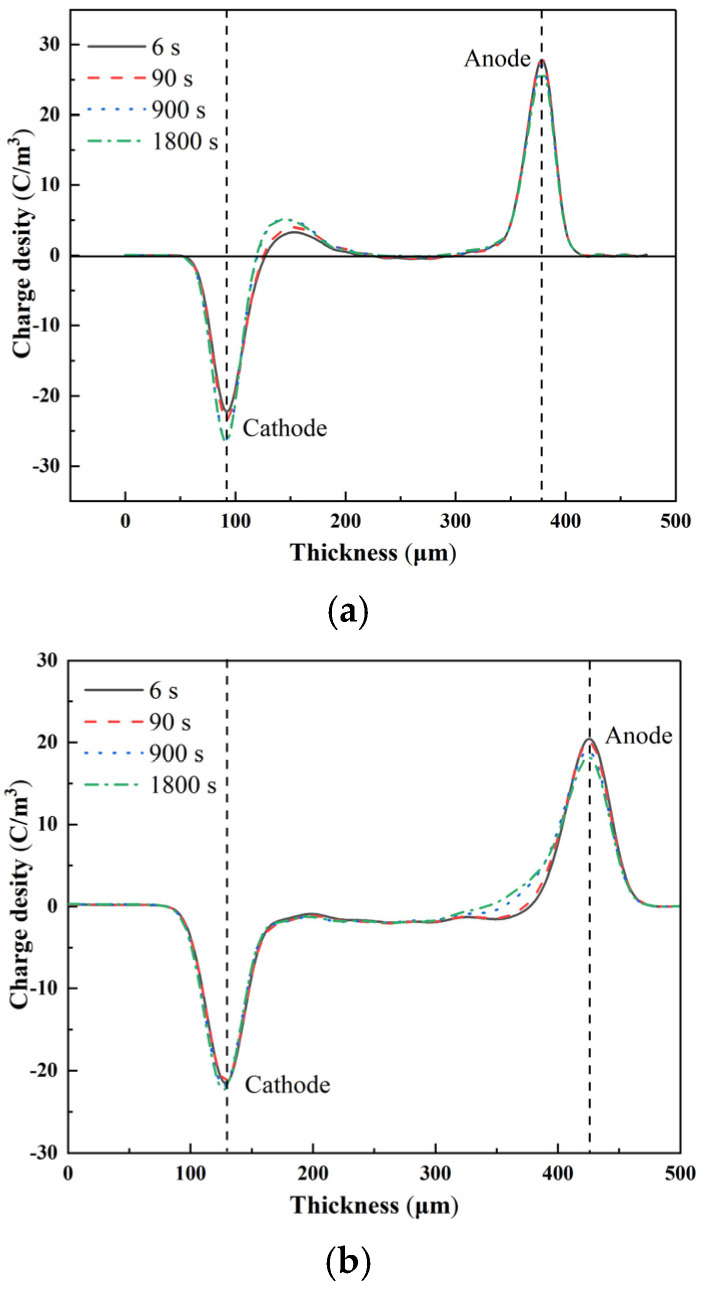

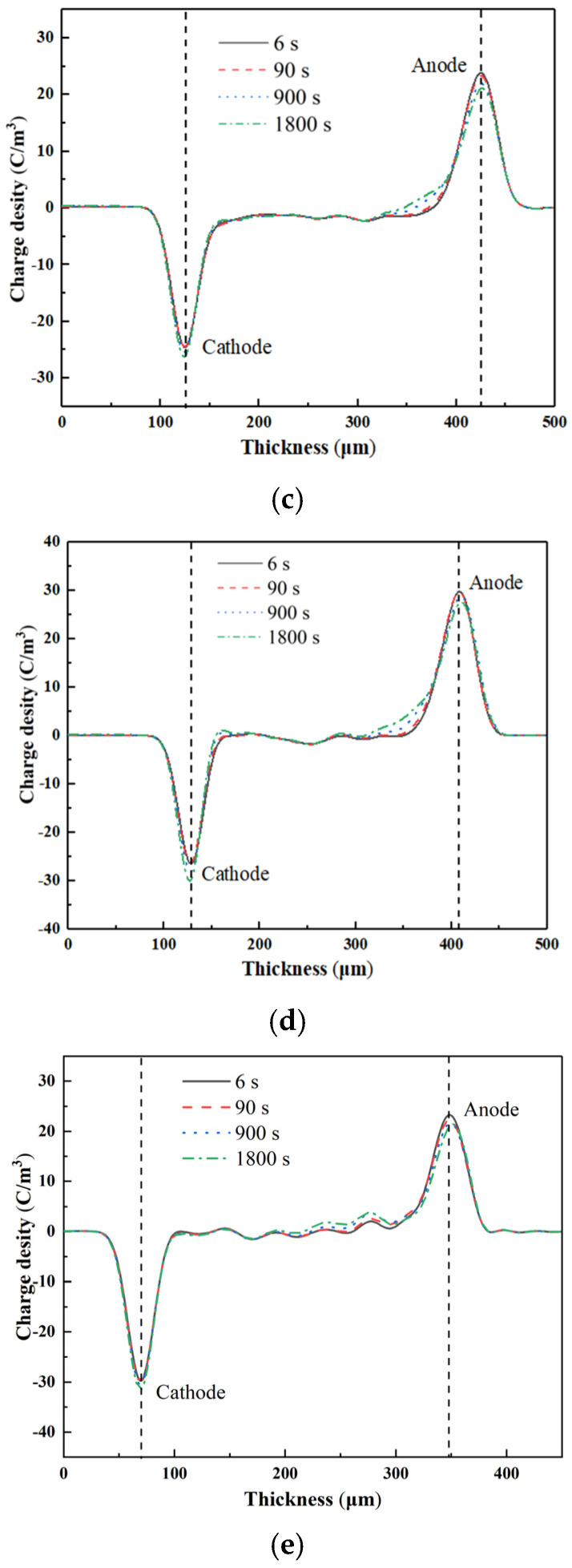
Space-charge distribution of pure LDPE and nanocomposite specimens with different content of Al_2_O_3_-h-BN/LDPE under 40 kV/mm polarization field: (**a**) LDPE; (**b**) 1% (0.5%h-BN/0.5%Al_2_O_3_)/LDPE; (**c**) 2% (1.0%h-BN/1.0%Al_2_O_3_)/LDPE; (**d**) 4% (2.0%h-BN/2.0%Al_2_O_3_)/LDPE; and (**e**) 7% (3.5%h-BN/3.5%Al_2_O_3_)/LDPE.

Figure 7 shows the space-charge distribution of the pure LDPE and Al_2_O_3_-h-BN/LDPE nanocomposite samples under a 40 kV/mm polarization field. Figure 7a shows that the pure LDPE formed an obvious accumulation of opposite polarity charges when pressurized for 6 s, and the positive polarity charges also gradually accumulated near the anode [19]. When the pressurization time reached 900 s, a large amount of space charge accumulated at the cathode to form a charge peak, and the positive charge of the anode decreased. At 1800 s, the charge peak near the cathode reached a saturation state with no significant change in charge accumulation, and the maximum charge density was 7.5 C/m^3^. Figure 7b–e show the space-charge distributions of the composite films with doping contents of 1 wt%, 2 wt%, 4 wt%, and 7 wt%, respectively. When the polarization was 6 s, the 1 wt% and 2 wt% composite films with a low doping amount had almost zero charge accumulation at the cathode and anode, and the space charge near the anode accumulated a little with increased polarization time, which was primarily due to the low trapping of space charges by deep traps brought about by the good dispersion of the two kinds of nanoparticles with doping content in the LDPE [20]. When the doping content was 4 wt%, a small amount of space-charge accumulation occurred at the cathode and anode when the polarization time was 6 s, and the anode was slightly more than the cathode. When the polarization time reached 1800 s, the cathode-charge accumulation increased compared with 900 s, but it was still low. Note that the space charge at the cathode did not increase significantly with time when the mixing concentration reached the maximum 7 wt%, and there were significant accumulations of charge packets at the anode.

**Figure 8:**
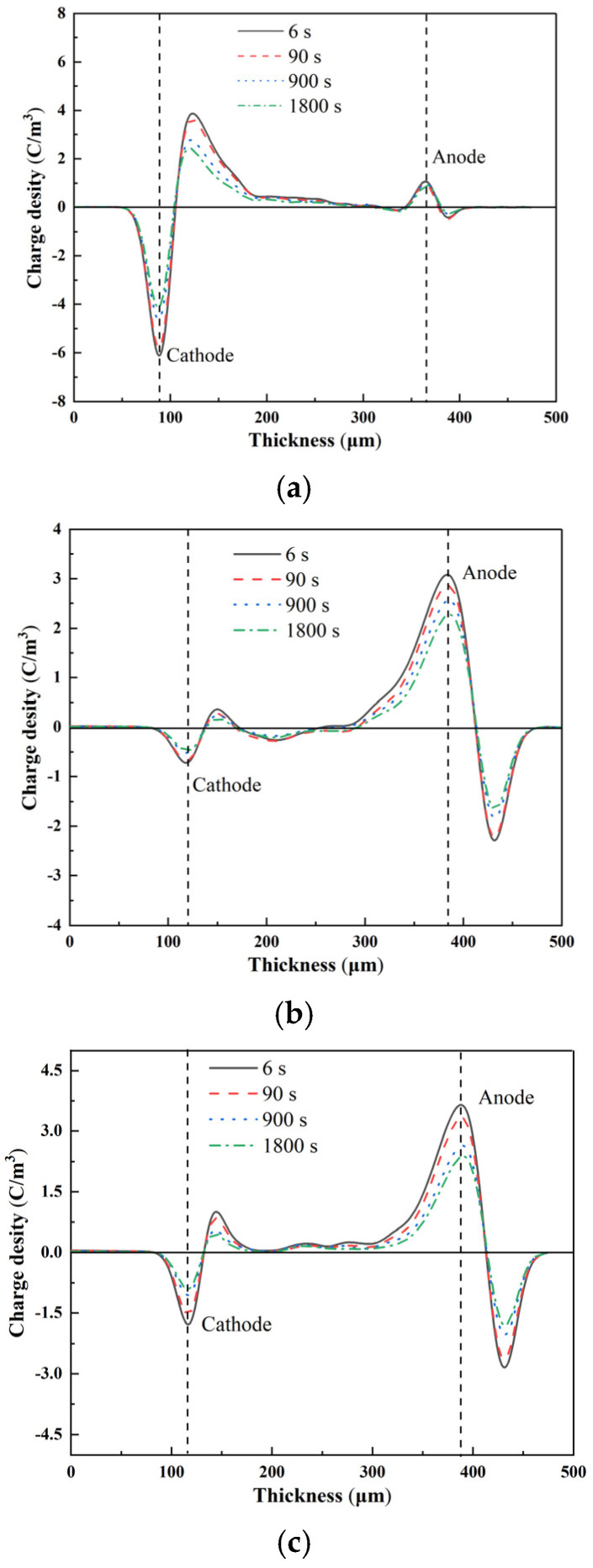

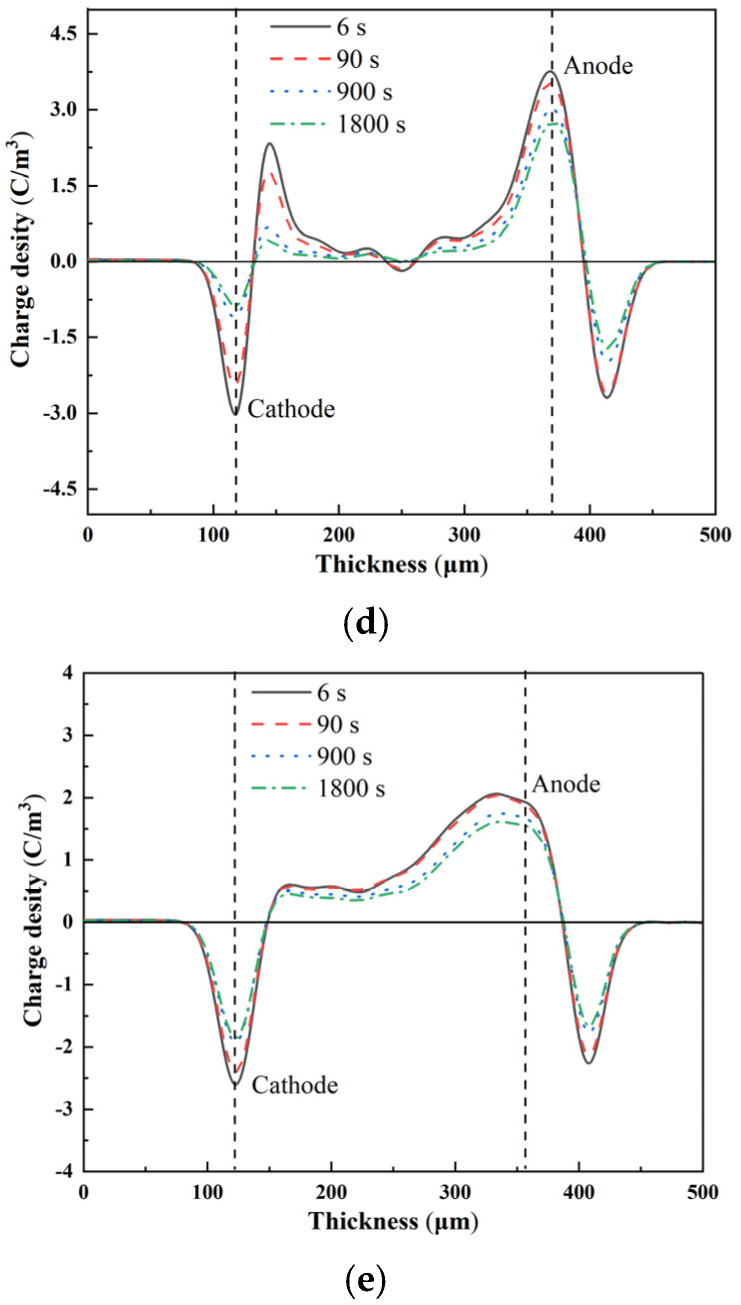
Space-charge distribution of pure LDPE and nanocomposite specimens with different contents of Al_2_O_3_-h-BN/LDPE in short circuit of 1800 s: (**a**) LDPE; (**b**) 1% (0.5%h-BN/0.5%Al_2_O_3_)/LDPE; (**c**) 2% (1.0%h-BN/1.0%Al_2_O_3_)/LDPE; (**d**) 4% (2.0%h-BN/2.0%Al_2_O_3_)/LDPE; and (**e**) 7% (3.5%h-BN/3.5%Al_2_O_3_)/LDPE.

Figure 8 shows the space-charge distribution of the pure LDPE and Al_2_O_3_-h-BN/LDPE nanocomposite samples within 1800 s of the short circuit [21]. As shown in Figure 8a, within the short-circuit time of 1800 s, a large positive charge-accumulation peak near the pure LDPE cathode gradually became smaller, which may have been due to the large concentration difference of the pure LDPE cathode. Thus, the charge peak decreased with time. Compared with the pure LDPE, the space charge of the composite film doped with two kinds of nanoparticles (h-BN and Al_2_O_3_) was significantly reduced, which was primarily due to the addition of nanoparticles during the pressurized polarization affecting the space charge in the composite film. The inhibition of the charge resulted in less space-charge accumulation [22]. The space-charge distribution diagrams of different nanodoping contents were 1 wt%, 2 wt%, 4 wt%, and 7 wt%. When the doped h-BN and Al_2_O_3_ were 1 wt% and 2 wt% and the short-circuit time was 6 s, the charge peaks of the cathode were only 0.36 and 1.04 C/m^3^, respectively. When the time reached 900 s, the space charge near the cathode decayed to 0.19 and 0.53 C/m^3^, respectively. When the time was 1800 s, the space charge near the cathode decreased compared with that at 900 s, but the attenuation was weaker. When doped with a higher content of nanofillers (4 wt% and 7 wt%), the space charge of the 4 wt% composite films had a large charge packet at about 90 s, and the charge packet gradually decayed to a small amount with increased depolarization time. Conversely, for the 7 wt% content of the composite film (the highest doping concentration), the nanoparticles were seriously agglomerated in the matrix, resulting in the excessive accumulation of space charges. The decay of space charges was the slowest during depolarization [23].

**Figure 9:**
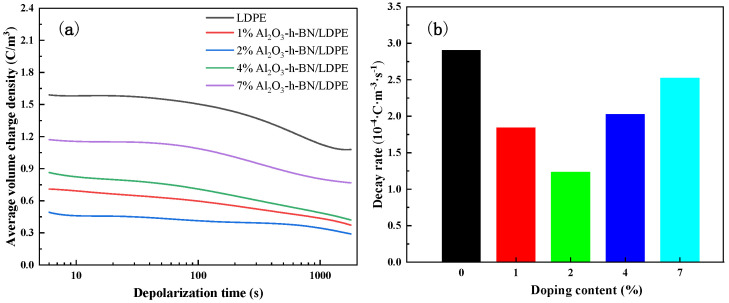
(**a**) Average volume-charge density and decay rate of LDPE and (**b**) its nanocomposite specimens during short circuit.

The average charge density and decay rate of the nanocomposite samples during the short-circuit process are shown in Figure 9a,b. Figure 9a shows that the average volume-charge densities of the films filled with nanocomposite samples were all smaller than that of the pure LDPE, and the average volume-charge density of the nanocomposite films doped with lower content was smaller than that of the composite films with higher content. When the doping content was 2 wt%, the average charge volume density was 0.48 C/m^3^ and was the lowest, that is, the effect of doping the appropriate nanoparticle concentration on the space charge was the optimum. Figure 9b also shows that when the appropriate nanofillers were doped, the decay rate during the short circuit was shortened owing to less space-charge accumulation and a small charge-density gradient difference. Meanwhile, the interface between the appropriate nanofiller and the matrix further suppressed the accumulation of space charges owing to the reverse electric field formed by the traps trapping space charges during polarization [24]. Space charge is known to exert an inhibitory effect [25]. In summary, the composite film with a doping content of 2 wt% had a better space-charge suppression effect, and the electrical performance was better when applied to high-voltage DC cables.

### 3.4. Thermal Conductivity Test

**Figure 10:**
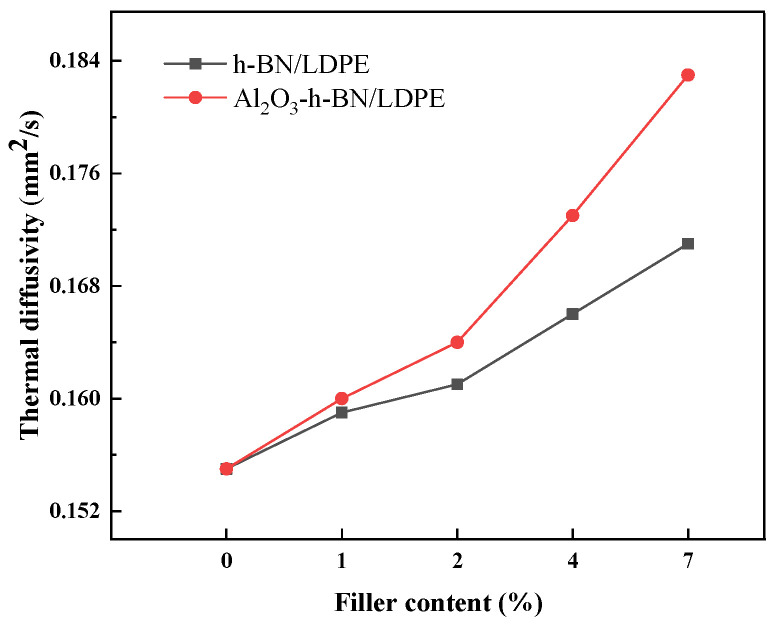
Thermal diffusion coefficients of h-BN/LDPE and Al_2_O_3_-h-BN/LDPE nanocomposite specimens with different doping contents.

Figure 10 is a graph of the thermal-diffusivity variation of single-doped nano-h-BN and mixed nano-h-BN and Al_2_O_3_ with doping amount. The thermal diffusivity of the Al_2_O_3_ fibers and h-BN particles mixed with two nanofillers at different concentrations was higher than that of single-doped h-BN nanofillers with a further increased concentration of doped nanofillers [26]. The thermal diffusivity of the composite film mixed with h-BN particles and Al_2_O_3_ fibers was much larger than that of single-doped h-BN. When the doping content was greater than 2 wt%, the thermal diffusivity of the two composite films was linear, but the thermal diffusivity curve of the composite film doped with the two fillers h-BN and Al_2_O_3_ fibers had a larger slope. When the fillers of the two composite films reached the maximum 7 wt%, the thermal diffusivity of the Al_2_O_3_-h-BN/LDPE composite film was 0.183 mm^2^/s, and the thermal diffusivity of the single-doped h-BN composite film was 0.172 mm^2^/s, both higher than that of the pure LDPE (0.155 mm^2^/s). The thermal diffusivity of the nanofiller composite films doped with the two types was 90.6% higher than that of the pure LDPE. This finding may be related to the heat transfer of inorganic fillers in polyethylene. Although nano-h-BN had a good thermal conductivity [27], the thermal conduction channels formed in the LDPE were limited, and Al_2_O_3_ had a good long-term performance [28]. The diameter ratio can form a synergistic effect with the h-BN particles in the thermal conduction channel, and further improve the thermal conductivity of the composite film [29].

**Figure 11:**
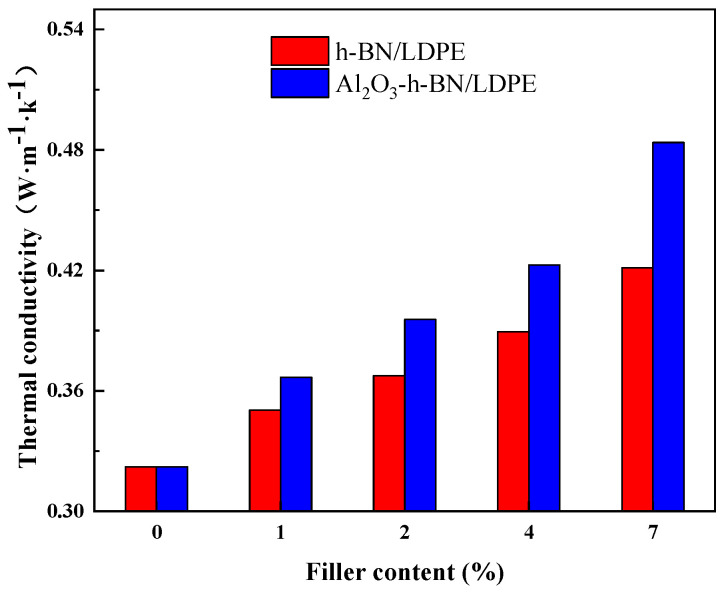
Thermal conductivity of h-BN/LDPE and Al_2_O_3_-h-BN/LDPE nanocomposite specimens with different doping contents.

Figure 11 shows the thermal conductivity comparison of the h-BN/LDPE and Al_2_O_3_-h-BN/LDPE nanocomposite films with different doping contents. The thermal conductivity of the pure LDPE was 0.32 W/(m·K). The thermal conductivity of nanocomposite films doping with single-doped (h-BN) and two-nanoparticle-doped (h-BN and Al_2_O_3_) fibers increases with the doping concentration of nanoparticles. However, under different nanoparticle contents, the thermal conductivity of h-BN and Al_2_O_3_ fibers doped with two kinds of nanoparticles in LDPE was higher than that of single-doped h-BN. When the nanodoping concentration reached 7 wt%, the thermal conductivity reached the maximum value. The thermal conductivity of Al_2_O_3_-h-BN/LDPE was 0.51 W/(m·K), and the thermal conductivity of h-BN/LDPE was 0.425 W/(m·K). Thus, compared with single-doped nano-h-BN particles, Al_2_O_3,_ and h-BN particles were more likely to form thermal conduction channels in the matrix [30], which greatly improved the thermal conductivity of the nanocomposite films. The thermal conductivity of the composite film doped with two kinds of nanoparticles into the LDPE was about 59.4% higher than that of the pure LDPE (whose thermal conductivity was 0.32 W/(m·K) at 7 wt% content). This thermal conductivity was 20% higher than that of the single-doped h-BN composite film. Therefore, the composite film mixed with nano-Al_2_O_3_ and h-BN had a better thermal conductivity and can be better used in high-voltage DC cables.

## 4. Conclusions

In summary, we prepared Al_2_O_3_-h-BN/LDPE composite materials with a high thermal conductivity and excellent electrical insulation properties by melt blending. The microstructure, dielectric properties, space charge properties, and thermal conductivity of the composite film were studied. It was found that the composite film doped with a small amount of nano-material h-BN particles and Al_2_O_3_ fibers had a good dispersion (i.e., 2 wt%) demonstrating a low dielectric constant, excellent space charge suppression, and high DC breakdown strength. In addition, the breakdown field strength was 12.3% higher than that of the pure LDPE and the thermal conductivity was about 23.8% higher than that of the pure LDPE. Therefore, adding a proper amount of h-BN and Al_2_O_3_ fillers can effectively improve the thermal conductivity and electrical properties of composite materials. This paper brings up new insight into the preparation of HVDC cable insulation materials.

## Figures and Tables

**Table 1:** The composition ratio of composite samples.

Sample	A (wt%)	B (wt%)	C (wt%)
LDPE	100	0	0
1 wt% Al_2_O_3_-h-BN/LDPE	99	0.5	0.5
2 wt% Al_2_O_3_-h-BN/LDPE	98	1.0	1.0
4 wt% Al_2_O_3_-h-BN/LDPE	96	2.0	2.0
7 wt% Al_2_O_3_-h-BN/LDPE	93	3.5	3.5

**Table 2:** Variation in Weibull distribution parameters of pure LDPE and its nanoparticle-doped composite specimens.

Sample	Quantity/N	Weibull Parameter	
		*E*_0_/(kV/mm)	β
LDPE	12	302.8	9.78
1 wt%Al_2_O_3_-h-BN/LDPE	12	319.6	9.07
2 wt%Al_2_O_3_-h-BN/LDPE	12	340.1	10.72
4 wt%Al_2_O_3_-h-BN/LDPE	12	278.2	9.26
7 wt%Al_2_O_3_-h-BN/LDPE	12	255.1	8.24

## References

[1] Chen G., Tu Y., Wang C., Li C. (2021). Environment-friendly Insulating Gases for HVDC Gas-insulated Transmission Lines. J. Power Energy Syst..

[2] He L.J., Zeng J.J., Huang Y.W., Yang X., Li D.W., Chen Y., Yang X.Y., Wang D.B., Zhang Y.X., Fu Z.D. (2020). Enhanced Thermal Conductivity and Dielectric Properties of h-BN/LDPE Composites. Materials.

[3] Zarubin V.S., Kuvyrkin G.N., Savelyeva I.Y. (2020). A Variation Model of Thermal Breakdown of a High-Voltage DC Cable Electrical Insulation. Russ. Electr. Eng..

[4] Zhang D.L., Zha J.W., Li C.Q., Li W.K., Wang S.J., Wen Y., Dang Z.M. (2017). High thermal conductivity and excellent electrical insulation performance in double-percolated three-phase polymer nanocomposites. Compos. Sci. Technol..

[5] Du B.X., Hang X. (2014). Effects of thermal conductivity on dc resistance to erosion of silicone rubber/BN nanocomposites. IEEE Trans. Dielectr. Electr. Insul..

[6] Zou D., Huang X., Zhu Y., Chen J., Jiang P. (2019). Boron nitride nanosheets endow the traditional dielectric polymer composites with advanced thermal management capability. Compos. Sci. Technol..

[7] Fei C., Liu Y.X. (2020). Preparation and Characterization of Al_2_O_3_ Heat Conduction Enhanced Polyimide Film. Fiber. Compos..

[8] Martinez-Oviedo A., Kshetri Y.K., Joshi B., Lee S.W. (2021). Surface modification of blue TiO_2_ with silane coupling agent for NOx abatement. Prog. Nat. Sci. Mater. Int..

[9] Suzuoki Y., Cai G.X., Mizutani T., Idea M. (1982). TSC Study on Interfacial Phenomena in PE (Polyethylene)-EVA (Ethylene-Vinylacetate Copolymer) Laminated Films. Jpn. J. Appl. Phys..

[10] Kim K., Kim M., Kim J. (2014). Enhancement of the thermal and mechanical properties of a surface-modified boron nitride–polyurethane composite. Int. J. Hydrogen. Energy.

[11] Shan F.R., Yu Z.M., Luo L.S., Zhang Y. (2013). Study on surface modification of nano-alumina by silane coupling agent KH550. New Chem. Mater..

[12] Reddy M.P., Manakari V., Parande G., Ubaid F., Shakoor R.A., Mohamed A.M., Gupta M. (2018). Enhancing compressive, tensile, thermal and damping response of pure Al using BN nanoparticles. J. Alloys Compd..

[13] Han S.J., Gross L.H. Electroluminescence test to evaluate dielectric property at the interface between semiconductive shield and insulation. Proceedings of the Conference Record of the 2004 IEEE International Symposium on Electrical Insulation.

[14] Huang X., Zhi C., Jiang P., Colberg D., Bando Y., Tanaka T. (2013). Polyhedral Oligosilsesquioxane-Modified Boron Nitride Nanotube Based Epoxy Nanocomposites: An Ideal Dielectric Material with High Thermal Conductivity. Adv. Funct. Mater..

[15] Chi Q., Ma T., Zhang Y., Chen Q., Zhang C., Cui Y., Zhang T., Lin J., Wang X., Lei Q. (2017). Excellent energy storage of sandwich-structured PVDF-based composite at low electric field by introduced the hybrid CoFe_2_O_4_@BZT-BCT nanofibers. Acs Sustain. Chem. Eng..

[16] Winell S., Annersten H., Prakapenka V. (2006). The high-pressure phase transformation and breakdown of MgFe_2_O_4_. Am. Mineral..

[17] Fan M., Zhang Y., Li X.J., Song H. (2020). High thermoelectric performance in nano-SiC dispersed Bi_1.6_Pb_0.4_Sr_2_Co_2_O_y_ compounds. J. Alloys Compd..

[18] Kovchavtsev A.P., Tsarenko A.V., Guzev A.A., Aksenov M.S., Polovinkin V.G., Nastovjak A.E., Valisheva N.A. (2015). The influence of electron energy quantization in a space-charge region on the accumulation capacitance of InAs metal-oxide-semiconductor capacitors. J. Appl. Phys..

[19] Mizutani T. Behavior of Charge Carriers in Organic Insulating Materials. Proceedings of the 2006 IEEE Conference on Electrical Insulation and Dielectric Phenomena.

[20] He L.J., Niu H.Q., Ma Y., Li D.W., Zhao L., Chen C.T. (2018). Effect of maleic anhydride on trap levels of alumina/low-density polyethylene by photo-stimulated discharge. Integr. Ferroelectr..

[21] Simmons J.G., Tam M.C. (1972). Theory of isothermal currents and the direct determination of trap parameters in semiconductors and insulators containing arbitrary trap distributions. Phys. Rev. B.

[22] Papathanassiou A.N., Sakellis I., Vitoratos E., Sakkopoulos S. (2017). Interfacial and space charge dielectric effects in Polypyrrole/Zinc Oxide composites. Synth. Met..

[23] Mitsumoto S., Nagao M., Fu M., Dissado L.A., Fothergill J.C. The space charge decay of high density polyethylene under different temperatures and accumulation fields. Proceedings of the 2007 IEEE International Conference on Solid Dielectrics.

[24] Huang X., Jiang P., Yin Y. (2009). Nanoparticle surface modification induced spacecharge suppression in linear low density polyethylene. Appl. Phys. Lett..

[25] Li S., Min D., Wang W., Chen G. (2016). Modelling of dielectric breakdown through chargedynamics for polymer nanocomposites. IEEE Trans. Dielectr. Electr. Insul..

[26] Wang Y., Wu J., Yin Y., Han T. (2020). Effect of micro and nano-size boron nitride and silicon carbide on thermal properties and partial discharge resistance of silicone elastomer composite. IEEE Trans. Dielectr. Electr. Insul..

[27] Liu Y., Li L., Liu H., Zhang M., Zhou S. (2020). Hollow polymeric microsphere-filled silicone-modified epoxy as an internally insulated material for composite cross-arm applications. Compos. Sci. Technol..

[28] Nakajima D., Kikuchi T., Natsui S., Sakaguchi N., Suzuki R.O. (2015). Fabrication of a novel aluminum surface covered by numerous high-aspect-ratio anodic alumina nanofibers. Appl. Surf. Sci..

[29] Naovaratpong S., Boonyaroonate I., Nathakaranakule A. (2014). Plasma Density Control in a Dielectric Barrier Discharge (DBD) Ozone Generator Using a Laser Engraved Dielectric Layer and Alumina Sand Filled Discharge Channel. Lasers Eng..

[30] Tian Z., Kim S., Sun Y., White B. (2009). A Molecular Dynamics Study of Thermal Conductivity in Nanocomposites via the Phonon Wave Packet Method. Proc. ASME InterPack Conf..

